# Advantages of a Training Course for Surgical Planning in Virtual Reality for Oral and Maxillofacial Surgery: Crossover Study

**DOI:** 10.2196/40541

**Published:** 2023-01-19

**Authors:** Max Ulbrich, Vincent Van den Bosch, Andrea Bönsch, Lennart Johannes Gruber, Mark Ooms, Claire Melchior, Ila Motmaen, Caroline Wilpert, Ashkan Rashad, Torsten Wolfgang Kuhlen, Frank Hölzle, Behrus Puladi

**Affiliations:** 1 Department of Oral and Maxillofacial Surgery University Hospital RWTH Aachen Aachen Germany; 2 Department of Diagnostic and Interventional Radiology University Hospital RWTH Aachen Aachen Germany; 3 Visual Computing Institute Faculty of Mathematics, Computer Science and Natural Sciences RWTH Aachen University Aachen Germany; 4 Institut of Medical Informatics University Hospital RWTH Aachen Aachen Germany

**Keywords:** virtual surgical planning, virtual reality, Elucis, 3D Slicer, oral and maxillofacial surgery

## Abstract

**Background:**

As an integral part of computer-assisted surgery, virtual surgical planning (VSP) leads to significantly better surgery results, such as for oral and maxillofacial reconstruction with microvascular grafts of the fibula or iliac crest. It is performed on a 2D computer desktop screen (DS) based on preoperative medical imaging. However, in this environment, VSP is associated with shortcomings, such as a time-consuming planning process and the requirement of a learning process. Therefore, a virtual reality (VR)–based VSP application has great potential to reduce or even overcome these shortcomings due to the benefits of visuospatial vision, bimanual interaction, and full immersion. However, the efficacy of such a VR environment has not yet been investigated.

**Objective:**

This study aimed to demonstrate the possible advantages of a VR environment through a substep of VSP, specifically the segmentation of the fibula (calf bone) and os coxae (hip bone), by conducting a training course in both DS and VR environments and comparing the results.

**Methods:**

During the training course, 6 novices were taught how to use a software application in a DS environment (3D Slicer) and in a VR environment (Elucis) for the segmentation of the fibula and os coxae, and they were asked to carry out the maneuvers as accurately and quickly as possible. Overall, 13 fibula and 13 os coxae were segmented for each participant in both methods (VR and DS), resulting in 156 different models (78 fibula and 78 os coxae) per method (VR and DS) and 312 models in total. The individual learning processes in both environments were compared using objective criteria (time and segmentation performance) and self-reported questionnaires. The models resulting from the segmentation were compared mathematically (Hausdorff distance and Dice coefficient) and evaluated by 2 experienced radiologists in a blinded manner.

**Results:**

A much faster learning curve was observed for the VR environment than the DS environment (β=.86 vs β=.25). This nearly doubled the segmentation speed (cm^3^/min) by the end of training, leading to a shorter time (*P*<.001) to reach a qualitative result. However, there was no qualitative difference between the models for VR and DS (*P*=.99). The VR environment was perceived by participants as more intuitive and less exhausting, and was favored over the DS environment.

**Conclusions:**

The more rapid learning process and the ability to work faster in the VR environment could save time and reduce the VSP workload, providing certain advantages over the DS environment.

## Introduction

Malignant or benign tumors, advanced osteomyelitis, osteoradionecrosis, and complex fractures can lead to extensive bone and soft tissue defects with the need for reconstruction. Therefore, soft and hard tissue reconstruction is an important and commonly used procedure in oral and maxillofacial surgery [1]. Microvascular reconstruction with fibula or iliac crest bone transplants is one of the best options for mandible bone reconstruction [2,3], and it has the highest success rate and delivers the best functional and esthetic results [4,5]. Microvascular reconstruction has been performed using a conventional technique, specifically manual free transplant raising, adjustment, and insertion [6,7].

Given its numerous clinical advantages, a virtual surgical planning (VSP) approach prior to microvascular reconstruction of the jaw has seen increased popularity. This approach involves using preoperative medical imaging within computer-assisted surgery (CAS) applications [8,9]. The advantages of VSP include reduced ischemia, a shorter defect reconstruction time, a shorter surgical procedure [10], a shorter length of hospital stay [10], a lower number of necessary osteotomy revisions, an overall lower volume of bone removed, a lower rate of osseous injury [11], and a better match of removed bone volume to defect volume [12].

However, VSP is associated with a higher preoperative workload [8] and is therefore often delegated to younger fellows or technical staff [13,14]. Despite this, VSP still remains an integral part of the surgical process and should be done or supervised by the performing surgeon [15]. Furthermore, learning VSP is time-consuming and requires an appropriate amount of learning time, but these investments are required to ultimately achieve good clinical outcomes [15-18].

One of the main bottlenecks is the preparation of 3D models by segmentation [19]. This process is still performed on a 2D computer desktop screen (DS) with 2D controls, such as a mouse and keyboard [20], which however seems unsuitable per se for such a task [21]. Furthermore, working in a DS environment differs from working in a surgical site since the DS environment lacks stereoscopic vision, and the use of a mouse and keyboard does not resemble working with surgical instruments at all. This leads to discrepancies between the VSP performed in a DS environment and the surgery performed in an operating theater.

A potential way to overcome the disadvantages of VSP performed in a DS environment could be the use of virtual reality (VR) [22] since it offers stereoscopic vision, allows the user to work manually, and allows more focused work due to immersion [21,23]. VR environments better resemble realistic work routines in the operating theater, as users can rotate and flip anatomical structures and observe different intraoperative viewing angles [24], thus enabling an enhanced understanding of the anatomy [25]. Although VR more broadly has been applied to surgery, it has often been basic or partially immersive VR. This must be distinguished from enhanced, fully immersive, binocular head-based VR [26]. With the so-called second wave that began in 2012, this technology has significantly spread in the consumer market and must be distinguished from augmented reality–based systems, which have become widespread under the terms mixed or extended reality [27]. Areas of application for these new VR head-mounted displays (HMDs) are surgical education, surgical training, and surgical planning [28]. VR simulations have been shown to lead to an improvement in surgical skills among subjects [29]. VR was also used for the visualization of medical images in radiology [22]. Recently, VR has been used for spatial understanding [30] or in the course of multiuser conferences during surgical planning [31]. However, to our knowledge, the potential for performing all steps or substeps of VSP using VR HMDs has not yet been explored.

This study aimed to demonstrate the possible advantages of a VR environment through a substep of VSP, specifically the segmentation of the fibula (calf bone) and os coxae (hip bone), which is typically applied in oral and maxillofacial surgery, by conducting a training course in both DS and VR environments and comparing the results.

## Methods

### Cases

We retrospectively selected 78 (13 cases for every 6 participants) planning computed tomography (CT) scans acquired between 2015 and 2020 at the University Hospital RWTH Aachen originally intended for VSP for the mandible, maxilla, or other viscerocranial sites for microvascular reconstruction. The CT scans were first scored by 2 trained radiologists based on image quality (1, good; 2, moderate; 3, poor), bone quality (1, good; 2, moderate; 3, poor), and artifacts (1, none; 2, moderate; 3, plenty), and from this, a total score (range 3-9) was then derived for each case and radiologist. A mean value was then calculated for each case based on the 2 radiologists’ total scores.

### Ethics Approval

The local ethics committee of the Medical Faculty of RWTH Aachen University approved our study (approval number EK 471/20). The experimental protocol was carried out in accordance with the guidelines set by the Declaration of Helsinki. Informed consent was obtained from all participants involved in the study.

### Study Design

A total of 6 novices in VSP (5 oral and maxillofacial surgery residents and 1 final-year dentistry student) took part in the study. Each participant’s age (mean 33.2, SD 2.6 years), gender (1 female, 5 males), surgical work experience (0-4 years), prior experience with computers, and prior experience with VR were recorded. Each participant performed a mental rotation test [32] before training to examine the influence of their baseline visuospatial ability on segmentation performance.

**Figure 1:**
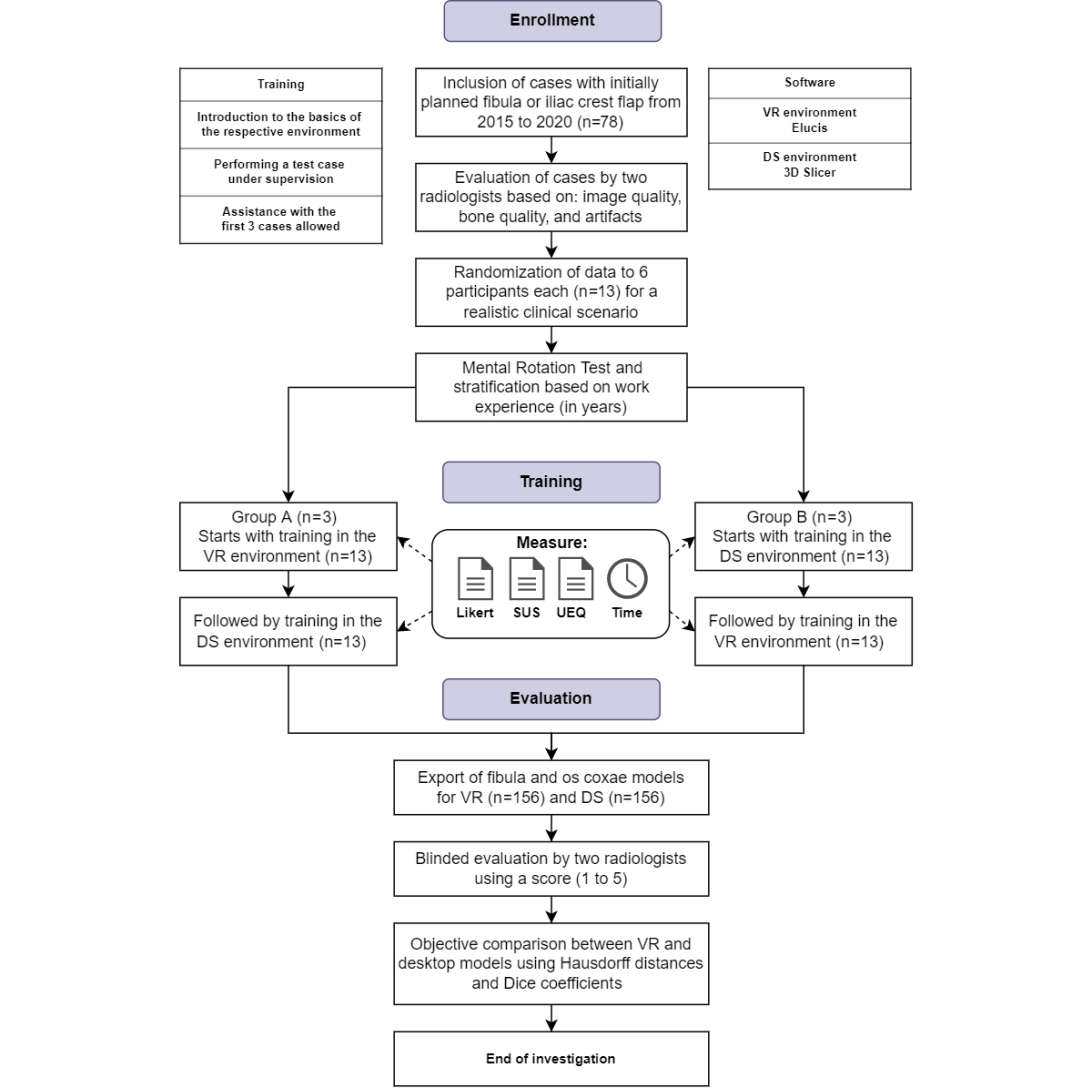
Graphic illustration of the study protocol. A total of 78 computed tomography scans were included, scored by 2 radiologists for image quality, and randomized to 6 participants, who additionally performed a mental rotation test before training. Subsequently, the 6 participants were stratified into Groups A and B, each of which started with the virtual reality (VR) or desktop screen (DS) environment, respectively. During the training, Likert-type questionnaires were filled out, and segmentation time was measured. After training, the System Usability Scale (SUS), the User Experience Questionnaire (UEQ), and a final Likert-type questionnaire were administered. After training, all fibula and hip bone models were compared using the Hausdorff distance and the Dice coefficient, and additionally evaluated in a blinded fashion by the same 2 radiologists.

All novices received an introduction to the basic principles of the VSP application. The participants were stratified into the following 2 groups based on surgical work experience: Group A (4 years, 3 years, and 0 years in residency) and Group B (4 years, 2 years, and 1 year in residency). Group A was trained with 13 (3 cases for initial warm-up and 10 cases for autonomous training) randomly selected (for a realistic clinical scenario) cases per participant, first in the DS environment and thereafter in the VR environment. In contrast, Group B was trained with 13 randomly selected cases per participant, first in the VR environment and thereafter in the DS environment (Figure 1). This ensured that a potential learning effect gained in one environment and transferred to the other was evenly distributed between both [18,33]. The given task of the training was the segmentation of the fibula (a simpler model) and os coxae (a more complex model) of the right side of the body in the corresponding working environment. The participants were asked to carry out their tasks as accurately and quickly as possible. In the case of relevant bone defects, the left side was used.

Each participant received an appropriate briefing in the corresponding working environment (DS or VR) with an explanation of all important functions, whereby the standard analog functions of both applications had to be used within a test case. Subsequently, the task was carried out based on a test case under the supervision of an experienced user who offered verbal guidance throughout each work step. The participants then started to work on the 13 cases. The opportunity to ask questions was allowed only for the first 3 cases to ensure a realistic clinical scenario. After each case, the procedure time was recorded (for fibula and os coxae segmentation, respectively), and a Likert-type questionnaire was completed [34]. All participants successfully completed the training. After completing the training for both environments, a final Likert-type questionnaire was completed. Additionally, for each application, the User Experience Questionnaire (UEQ) [35] and System Usability Scale (SUS) [36] were administered.

**Figure 2:**
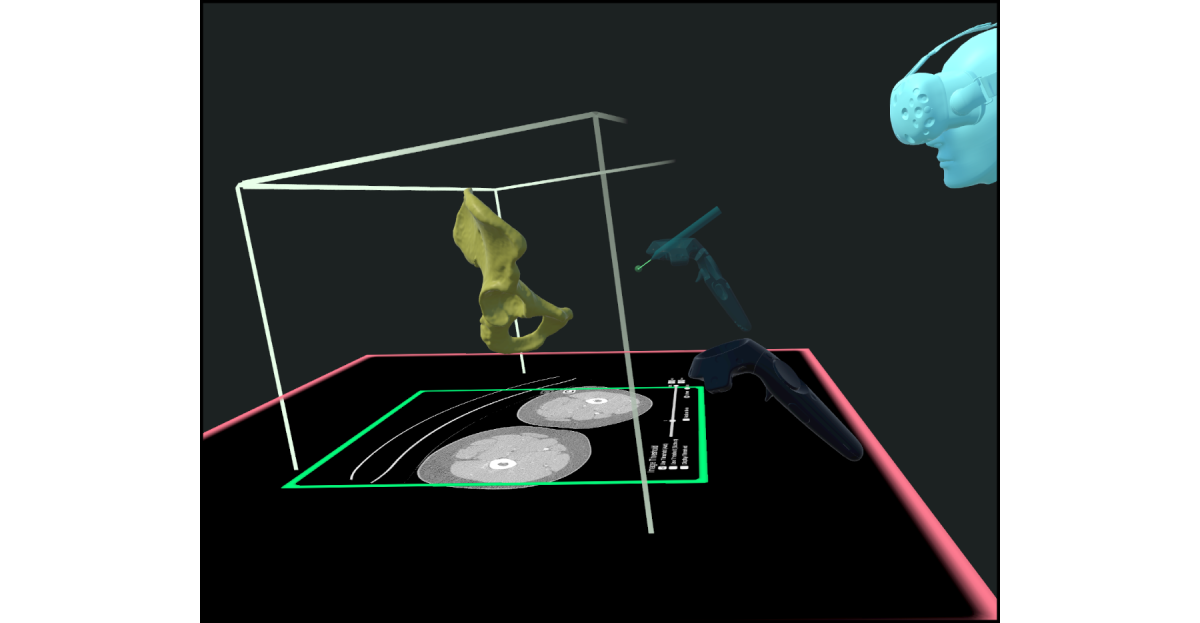
The virtual reality working environment (Elucis) and a segmented hip bone model in yellow in the middle.

**Figure 3:**
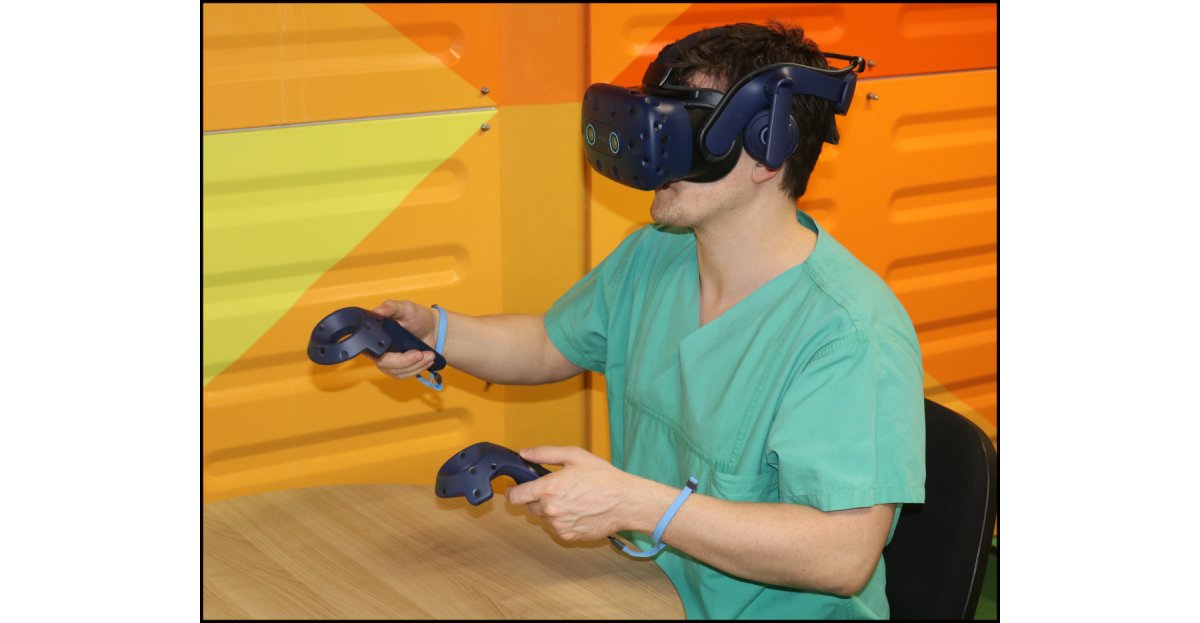
The virtual reality working environment from a third-person perspective.

**Figure 4:**
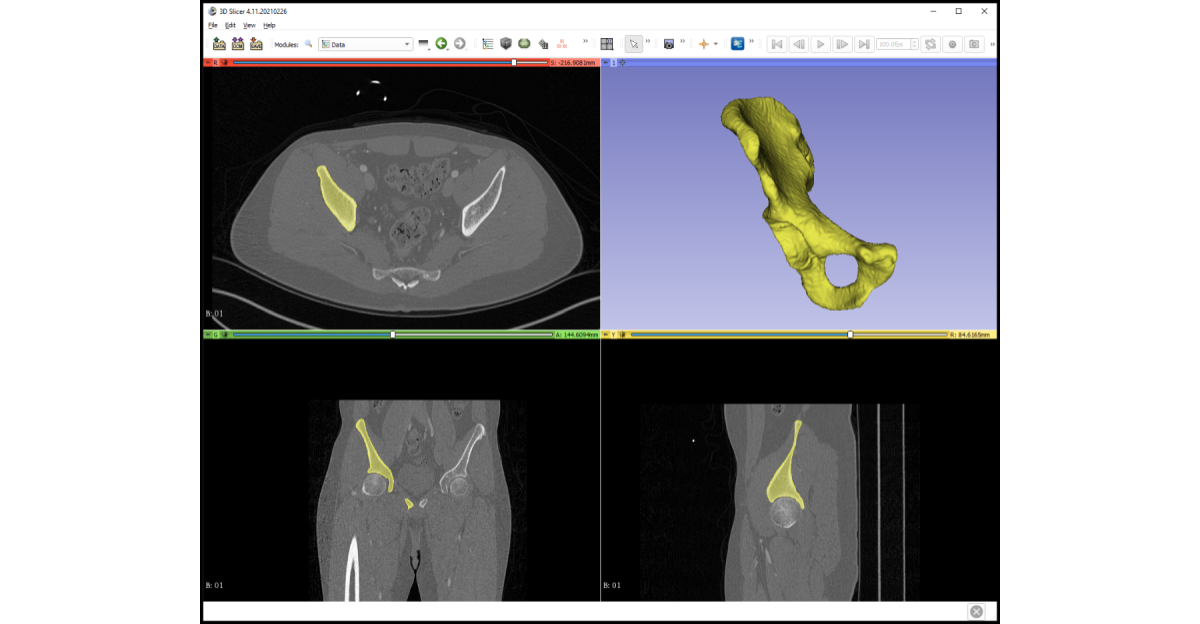
The classic desktop screen working environment (3D Slicer) with an already segmented os coxae (hip bone) model in yellow.

**Figure 5:**
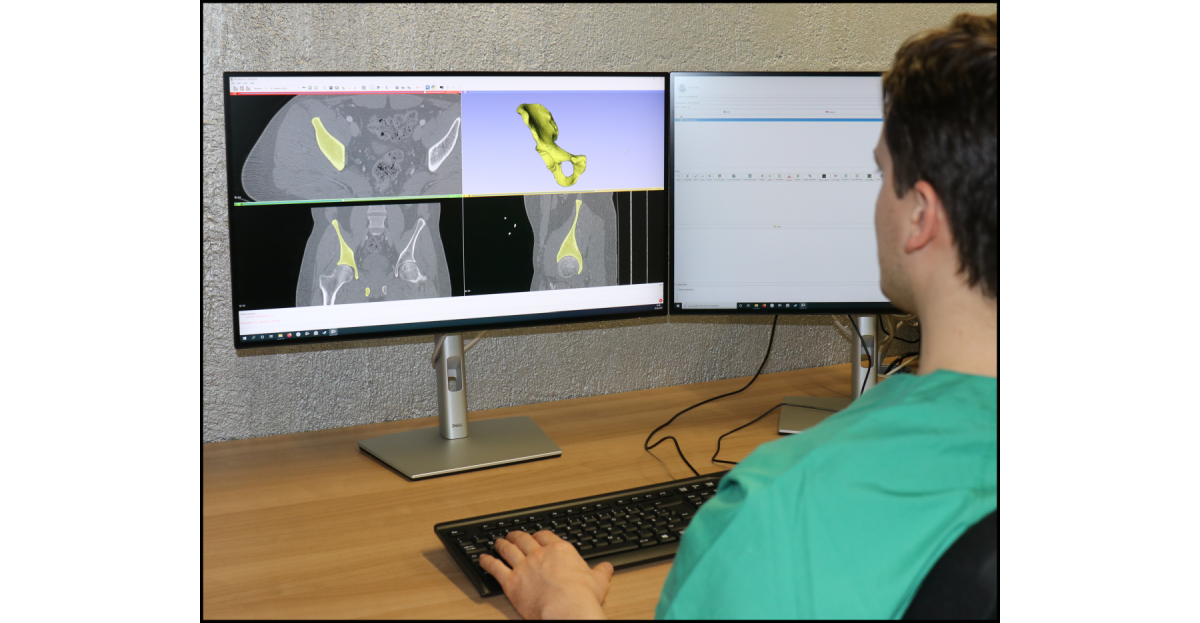
The desktop screen working environment from a third-person perspective.

DS-based training was performed using 3D Slicer version 4.11.20210226 [37], and VR-based training was performed using Elucis version 1.4 (Realize Medical Inc). The VR hardware included an HTC Vive Pro with an HTC Vive Controller 2.0 (Valve Corporation). All training was done seated (Figures 2-5) at the same workstation (AMD Ryzen 3900X CPU with 64 GB of memory and an RTX 2080 Ti graphics card).

### Evaluation

All segmented models of the fibula and os coxae (n=156) were assessed in a blinded setting by the same 2 trained radiologists using an absolute category rating (1, excellent; 2, good; 3, fair; 4, poor; 5, bad). Based on the evaluation of both radiologists, a mean value was calculated.

Afterwards, the postprocessing of all cases was performed using the 3D Slicer add-on Surface Wrap Solidify [38] to remove cavities in the models. This was necessary to avoid bias when comparing the volumes or surfaces of the models. Hausdorff distances and Dice coefficients [39] were then computed using the 3D Slicer add-on segment comparison [40] between the DS model and the VR model. Finally, intersection volumes (cm^3^) between the VR and DS models were divided by segmentation duration (minutes) to calculate common segmentation performance (cm^3^/min).

### Statistical Analysis

Statistical analysis was performed using the programming language R (Version 4.1.1; R Foundation for Statistical Computing). A *P* value <.05 was considered significant. We used a *t* test, a Wilcoxon signed-rank test, or a chi-square test to assess differences between VR and DS. The training effect as a function of the environment was determined as the duration of task completion using a linear mixed effect model with the R package lme4. For the model-based evaluations, a likelihood ratio test of the corresponding parameters was used to evaluate the relationships between dependent and independent variables. *P* values were adjusted for multiple testing using the Holm-Bonferroni method [41]. The 95% CIs were calculated by conventional bootstrapping with 1000 replications [42]. Plots were visualized using the R package ggplot2.

## Results

### Segmentation

The average overall radiologist ratings for the segmentation results were 1.40 (1, excellent; 5, bad) for the VR models and 1.29 for the DS models, and were not significantly different (Wilcoxon signed-rank test, *P*=.29). Similarly, there was no significant difference in radiologist assessment for the os coxae (VR 1.62 vs DS 1.39; Wilcoxon signed-rank test, *P*=.14) or fibula (VR 1.17 vs DS 1.17; Wilcoxon signed-rank test, *P*=.99; Table 1). Furthermore, regarding clinical use (models with a score ≤2/good), there was no relevant difference between VR and DS for all models, os coxae models, and fibula models (χ^2^, *P*=.99, *P*=.99, and *P*=.99, respectively). Thus, the VR models are equally suitable for clinical use as the DS models.

**Table 1:** Results of metric evaluations and blinded assessments by 2 radiologists.

Model and characteristics (n=312)			VR^a^ environment, mean (SD)		DS^b^ environment, mean (SD)		Difference, mean (SD)
**Os coxae (hip bone)**							
	Bone volume (cm^3^)	341 (65)		329 (62)		12 (12)	
	Segmentation duration (min)	22.5 (13.0)		38.7 (22.6)		−16.2 (21.3)	
	Segmentation performance (cm^3^/min)	19.2 (12.8)		12.0 (8.6)		7.2 (4.2)	
	Segmentation quality (range 1-5)	1.62 (0.76)		1.39 (0.63)		0.23 (0.78)	
	Hausdorff distance (mm)	N/A^c^		N/A		0.43 (0.19)	
	Dice coefficient (%)	N/A		N/A		0.96 (0.02)	
**Fibula (calf bone)**							
	Bone volume (cm^3^)	60 (12)		58 (11)		2 (3)	
	Segmentation duration (min)	12.1 (8.0)		17.0 (11.1)		−4.9 (8.3)	
	Segmentation performance (cm^3^/min)	6.5 (2.9)		4.7 (4.0)		1.8 (1.1)	
	Segmentation quality (range 1-5)	1.17 (0.38)		1.20 (0.44)		−0.03 (0.52)	
	Hausdorff distance (mm)	N/A		N/A		0.29 (0.12)	
	Dice coefficient (%)	N/A		N/A		0.96 (0.02)	

^a^VR: virtual reality.

^b^DS: desktop screen.

^c^N/A: not applicable.

**Figure 6:**
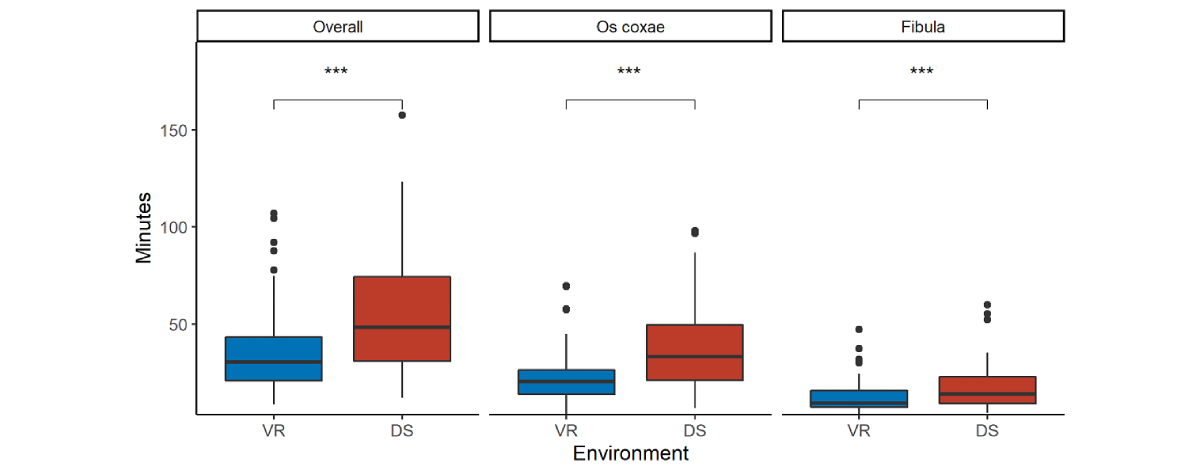
Comparison of segmentation time in minutes between the virtual reality (VR) environment (Elucis) in blue and the desktop screen (DS) environment (3D Slicer) in red shown as box-and-whisker plots. Box-and-whisker plots: boxes represent the IQR, thus representing 50% of data (Q1-Q3); the lower whisker is defined as Q1 − 1.5 × IQR; the upper whisker is defined as Q3 + 1.5 × IQR; the horizontal line in the middle of the box represents the median; and points are outliers. Additionally, the significance level of the respective Wilcoxon signed-rank test is shown above. The y-axis shows the segmentation time in minutes, and the x-axis shows the environment (VR vs DS). ***P<.001.

In contrast, the segmentation results from the VR environment were considered better than those from the DS environment by the participants themselves for both the os coxae models (7-point Likert scale [1, strongly disagree; 7, strongly agree], VR 5.5 vs DS 4.0) and fibula models (7-point Likert scale, VR 5.8 vs DS 5.1; Table 2). For the os coxae, the mean Hausdorff distance between VR and DS was 0.43 (SD 0.19) mm with a Dice coefficient of 96% (SD 2%). For the fibula, the mean Hausdorff distance between VR and DS was 0.29 (SD 0.12) mm with a Dice coefficient of 96% (SD 2%) (Table 1). The mean segmentation time of the os coxae models was 22.5 (SD 13.0) minutes for VR and 38.7 (SD 22.6) minutes for DS, and that of the fibula models was 12.1 (SD 8.0) minutes for VR and 17.0 (SD 11.1) minutes for DS (Figure 6; Table 1).

**Table 2:** Results of the Likert-scale questionnaire after each of the 13 training cases.

Likert (7-point) questions	Score in the VR^a^ environment (n=78), mean (SD)	Score in the DS^b^ environment (n=78), mean (SD)	Difference, mean (SD)
I learned something through this segmentation process.	5.7 (1.2)	4.9 (1.2)	0.8 (1.2)
I feel exhausted after the segmentation process.	4.4 (1.5)	5.2 (1.5)	−0.8 (1.8)
I had to repeat steps during segmentation.	4.3 (1.6)	5.0 (1.6)	−0.7 (1.6)
I consider this fibula to be adequately segmented.	5.8 (1.1)	5.1 (1.2)	0.7 (1.2)
I find this iliac crest to be adequately segmented.	5.5 (1.1)	4.0 (1.6)	1.5 (1.6)
I was able to orient myself spatially well during segmentation.	6.3 (0.7)	4.4 (1.3)	1.9 (1.7)

^a^VR: virtual reality.

^b^DS: desktop screen.

### Training Curve

**Figure 7:**
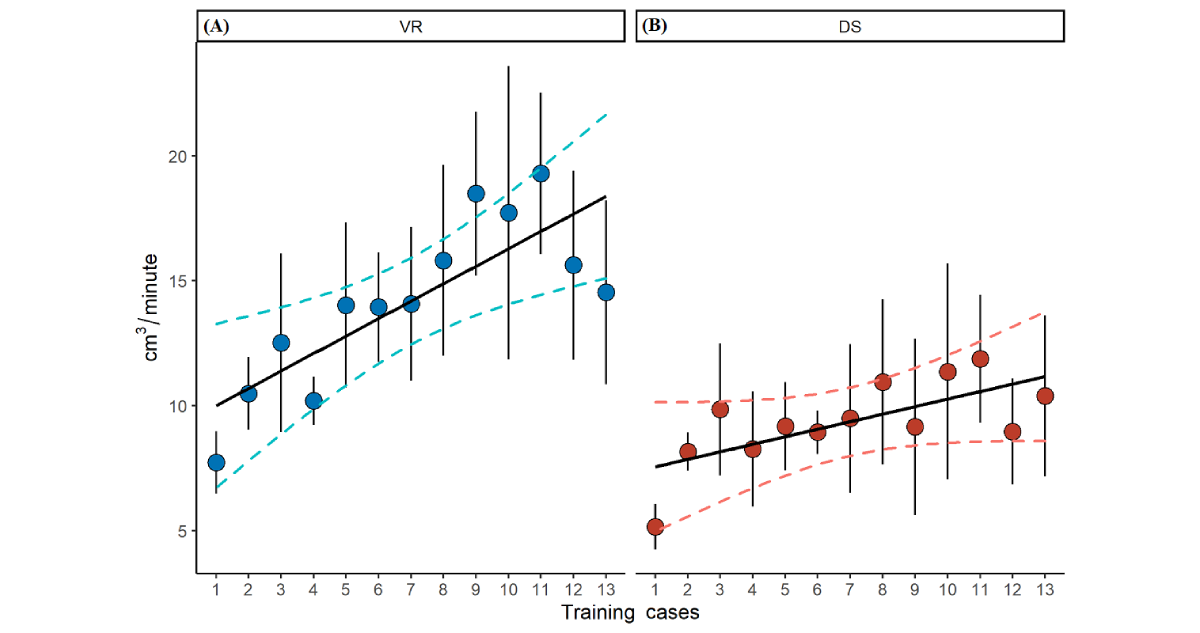
The segmented volume (cm3) per minute over the course of the 13 training cases in the (A) desktop screen (DS) environment and (B) virtual reality (VR) environment. The error bars represent the standard error of the mean, and the individual points are the mean values for the corresponding training case. The black line represents a linear model, and the dashed lines represent the 95% CIs of the model.

The linear mixed effect model (adjusted for work experience, mental rotation test, CT quality, segmentation quality, and training group) showed a significant increase in segmentation volume per minute (cm^3^/min) for the VR environment (β=.86; *P*<.001). While the training effect for the DS environment was not significant anymore after *P* adjustment (β=.25; *P*=.26; Figure 7). Lower CT quality had no influence on the segmentation process (*P*=.25). Segmentation performance was significantly higher in the VR environment than in the DS environment for the os coxae models (mean 19.2, SD 12.8 vs mean 12.0, SD 8.6 cm^3^/min; paired *t* test, *P*<.001) and for the fibula models (mean 6.5, SD 2.9 vs mean 4.7, SD 4.0 cm^3^/min; paired *t* test, *P*<.001; Table 1). These results correlated with the results of the Likert-type questions concerning the learning effect, exhaustion, and the perceived need to repeat steps (Table 2).

### Environment

**Figure 8:**
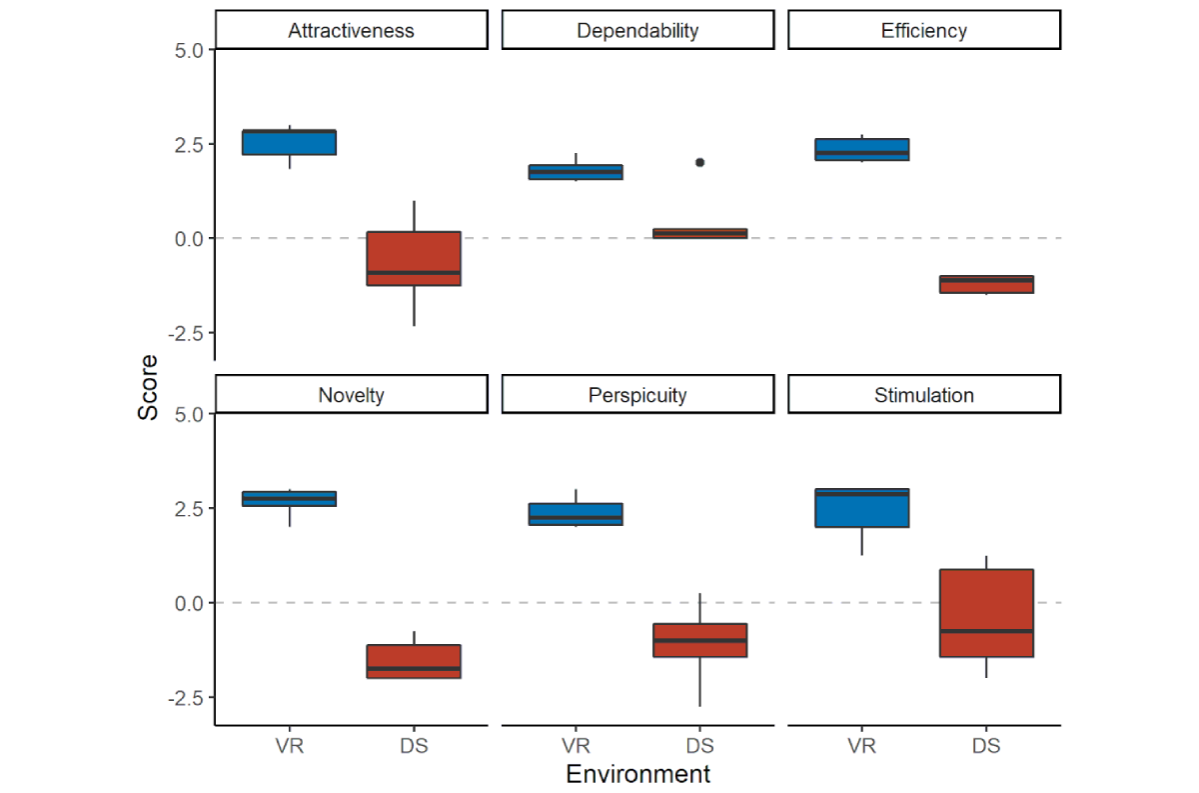
User experience questionnaire (UEQ) comparison between the virtual reality (VR) environment (Elucis) in blue and the desktop screen (DS) environment (3D Slicer) in red shown as box-and-whisker plots. Box-and-whisker plots: boxes represent the IQR, thus representing 50% of data (Q1-Q3); the lower whisker is defined as Q1 − 1.5 × IQR; the upper whisker is defined as Q3 + 1.5 × IQR; the horizontal line in the middle of the box represents the median; and points are outliers. The Y-axis shows the UEQ score, and the X-axis shows the environment (VR vs DS). A total of 6 categories were examined (attractiveness, dependability, effectiveness, novelty, perspicuity, and stimulation).

Overall, the participants clearly preferred the VR environment over the DS environment in a poststudy questionnaire (Tables 3 and 4). The UEQ results showed that the participants assessed the VR environment to be better in terms of attractiveness, dependability, efficiency, novelty, perspicuity, and stimulation (Figure 8). The SUS results showed that Elucis was rated 83.3 (95% CI 75.3-90.8) and 3D Slicer was rated 30.4 (95% CI 20.1-38.3).

**Table 3:** Results of the Likert-scale questionnaire after completion of training.

Likert (7-point) questions	Score in the VR^a^ environment (n=6), mean (SD)	Score in the DS^b^ environment (n=6), mean (SD)	Difference, mean (SD)
Segmentation in this environment was easy for me.	6.5 (0.5)	3.8 (1.9)	2.7 (2.0)
I would prefer this environment for VSP^c^.	7.0 (0.0)	2.5 (0.5)	4.5 (0.5)
This work environment seems intuitive to me.	6.8 (0.4)	2.3 (1.0)	4.5 (1.0)
I suspect this work environment will continue to be the gold standard in clinical practice for VSP.	6.7 (0.8)	2.2 (0.8)	4.5 (1.4)
Monoscopic (DS) or stereoscopic (VR) vision in this environment made segmentation easy for me.	6.7 (0.5)	2.2 (1.2)	4.5 (1.5)
I prefer learning segmentation with a mouse and keyboard (DS) or VR controller (VR).	6.7 (0.5)	2.3 (0.8)	4.3 (1.2)
I found learning segmentation easy in this environment.	6.3 (0.5)	3.2 (1.2)	3.2 (1.2)
I felt the training scheme was appropriate for learning in this environment.	6.2 (0.8)	5.7 (1.5)	0.5 (1.5)
I felt the number of cases to learn segmentation was sufficient in this environment.	6.3 (0.8)	5.3 (1.2)	1.0 (1.7)
I had fun while learning segmentation in this environment.	7.0 (0.0)	3.8 (1.9)	3.2 (1.9)

^a^VR: virtual reality.

^b^DS: desktop screen.

^c^VSP: virtual surgical planning.

**Table 4:** Comments of the participants comparing the virtual reality and desktop screen environments.

Participant No.	What aspects of the VR^a^ environment do you prefer over the DS^b^ environment?	What aspects of the DS environment do you prefer over the VR environment?
1	Spatial vision, working with controllers, similarity to OP site.	Plausibility (you don’t have to take off the HMD), work more precisely with the mouse at times, other applications usable.
2	Similarity of actions to surgical activity, stereoscopic vision, good spatial and anatomical orientation.	Island tool.
3	3D perception, rotating and handling the dataset in your own hands, direct segmentation in rendered state; you can directly see the result of segmentation without segmenting in 2D layers.	The possibility of labeling each voxel separately in each layer, if desired; thus, higher accuracy is possible.
4	Intuitive handling, spatial representation, fast learning progress.	Conventional desktop workplace.
5	Very intuitive in comparison, optimal spatial orientation, orientation also with regard to the future operation area, faster learning.	It was possible to use “ADD” function in difficult cases (e.g. calcified vessels in direct neighborhood); calcified vessels and bones often have similar Hounsfield units and cannot be visualized separately in Elucis.
6	Easier to learn, 3D environment easier to control, better spatial overview, much better control.	Lower acquisition costs.

^a^VR: virtual reality.

^b^DS: desktop screen.

## Discussion

VSP in a traditional DS environment is still considered the gold standard, despite its disadvantages, such as being tedious and time consuming [43]. This negates the benefit of time saved in the operating theater due to additional time spent on VSP [13,14]. In this context, VR is a promising approach that might attenuate the mentioned disadvantages of VSP performed in a DS environment.

In this study, the processes of learning VSP, using segmentation as a surrogate parameter, between traditional DS and fully immersive VR environments were compared, as segmentation is a common substep in VSP, a bottleneck in terms of time [19,44], and a crucial factor that determines the accuracy of the final computer-aided design (CAD) result [45]. In general, segmentation is important for not only oral and maxillofacial surgery but also many other medical specialties that require 3D models created by segmentation for the subsequent steps in CAD [45]. Therefore, segmentation was used as a surrogate parameter to measure performance in VR compared to performance in a traditional DS environment during a structured training course. For a representative number of cases (n=156), all participants had to create models of the fibula and os coxae, which are commonly used for microvascular reconstruction of the mandible [2,3], in both environments.

Many studies have shown that VSP is beneficial to patients [14,46]. However, VSP is only feasible if the surgeon is proficient in VSP software. For this reason, the process of learning how to operate such CAS-related software has already been the subject of research in the literature. For example, learning curves upon repetition have been found for VSP for the treatment of orbital fractures or in orthognathic surgery [15,47]. In addition, differences in learning curves when it comes to CAD programs used in dentistry have been found between different software and professional groups (students, technicians, and dentists) [18,33]. Furthermore, the learning curve has been shown to be a function of the complexity of the task for model creation using CAD [48]. However, all these studies used applications in a 2D DS environment, where a computer mouse and keyboard were utilized. This type of work environment is counterintuitive to surgeons who are accustomed to ambidextrous, manual, and visuospatial activity while being very focused. In contrast, VR is more similar to surgical work and therefore offers a promising approach.

The results of our study showed that working in a VR environment is significantly more efficient than working in a DS environment. At the same time, VR has a much faster learning curve (Figure 7). To have a comparable measure between models with different volumes, we used segmentation volume per minute (cm^3^/min), assuming that it significantly reflects the number of attempts to solve the task during the acquisition of cognitive and motor skills [48]. At the end of the training program, this was almost twice as high in the VR environment compared to the DS environment. To account for a transfer effect from one method to the other due to the crossover study design, we split the groups in correspondence to similar studies [18,33] and adjusted our model for training method order. To exclude bias from the multiple steps of the VSP as described in other studies that have evaluated the learning process for an entire VSP/CAS based on the final surgical outcome [15,49], we focused on the process performance itself. The measurable stronger learning effect in the VR environment was also perceived by the participants (Table 2). This is important, as the faster learning of CAD software, such as that for VSP, is also accompanied by increased planning and surgical accuracy, and improved anatomical understanding of the target structures [15,47]. Independent of segmentation performance, it is assumed that for different DS software, with correspondingly longer training periods, the differences will eventually cease to exist [33]. However, this does not explain the large differences observed between the DS and VR environments.

These differences can be explained by the principally different natures of the DS and VR environments. Subjects in our study reported a high degree of anatomical orientation in the VR environment compared to the DS environment (*P*<.001). One reason for the superior anatomical orientation might be the possibility of obtaining different viewing angles in the VR environment. Specifically, Cha et al demonstrated this advantage [24]. Furthermore, in our study, subjects preferred to work with both hands using 2 VR controllers, appreciated the advantages of stereoscopic vision, and preferred the intuitive perception provided by the VR environment, which might explain their preference for the VR environment over the DS environment overall (Table 3). In summary, this study highlighted the benefits of spatial perception and working in 3D in a VR environment with good orientation in terms of the anatomy and strong similarity to surgical work. Similar results have been found in the use of nonfully immersive VR for CAS of the liver [21]. In the literature, various approaches to using at least nonfully immersive VR in the medical context can be found. For example, stereoscopic monitors have been used in oral and maxillofacial surgery to determine facial soft tissue or orbital volumes [50,51]. Furthermore, they have been used in neurosurgery to train surgeons to resect intracerebral tumors [52] and assess surgical risk as a rehearsal before surgeries [25,53].

Yet, all of the aforementioned approaches lacked the full immersion that comes with using VR HMDs and controllers in both hands, as in our study. In our setting, users held control elements in both hands and worked with them within arm’s reach (Figures 2 and 3). This could be another reason for the rapid learning and efficiency of working in VR, as proprioception can then be used. Therefore, subjects felt particularly comfortable, and the highest precision could be achieved [54]. In contrast, working with a computer mouse and keyboard in the DS environment does not allow this. For complex models (os coxae) in comparison to simpler models (fibula), this advantage was even more pronounced in VR than in DS. In addition to the advantages of the stereoscopic visualization of complex models in VR [55], the reasons for this are that complex models require more commands for their implementation [48] and, at the same time, bimanual work in VR is particularly faster [56].

Regarding the segmentation quality of the os coxae model, a certain discrepancy between the radiologists’ evaluations and that of the subjects appeared to be a shortcoming, as the subjects tended to overestimate themselves. However, this had no impact on clinical practice, since with a given clinically relevant score of ≤2 (good or excellent), we found no significant difference between the 2 environments. A possible reason for this overestimation could be that in the VR environment by default, usually only 1 slice (axial, coronal, or sagittal) was displayed. In contrast, all 3 slices were displayed in parallel in the DS application. One solution would be to display all 3 slices by default in VR. In addition, a different perception of the 3D models (stereoscopic during segmentation by participants vs monoscopic during evaluation by radiologists) could have influenced this. Another reason could be the resolution capacity of the used VR HMD, causing individual voxels to be blurred. Improved hardware or better delineation of individual voxels in the single-layer view could probably solve this.

Overall, our results suggest that the intrinsic feedback learning effect of VSP, which a surgeon achieves by multiple repetitions of VSP and consecutive surgeries [15], could potentially be at least partially replaced by a training program in this manner for novices in VSP. Therefore, the results of our study suggest that VR should be the environment of choice, given its similarity to actual surgical activity. Furthermore, subjects consistently reported less fatigue and more enjoyment while working in the VR environment (Tables 2 and 3). This was also in line with the SUS results, which clearly rated VR better than DS. The reduced exhaustion can be explained by the shorter time needed to complete the task and possibly by the manual 3D interactions in VR, resembling the surgical working environment more realistically (Table 2). Participants perceived the VR software to be superior to the DS software according to the UEQ results for multiple qualities of experience (Figure 8) and preferred the VR environment over the DS environment (Table 3). This was in line with the results of a study on VR user interfaces for medical marking on 3D models [57]. Yet, these differences are surprising, since both applications basically fulfill the same task.

Experiencing the burden of today’s required levels of documentation, surgeons have long been demanding to spend less time at the computer [58]. This could probably also explain the poor rating for the DS environment, which is similar to the typical unpopular work on a DS computer. However, VR has a more playful character, which might explain the higher ratings for the VR environment. However, this clear difference in rating also shows that VSP applications should be adapted to the needs of surgeons. VR seems to meet these requirements, at least in the case of our test persons. In this context, the following things should be considered. Using a VR environment can lead to VR sickness. However, this was not reported by any of the participants, probably because they were sitting and no dynamic scenes were used [59]. In addition, it should be considered that older adults take longer to adapt when learning new technologies [60]. In this regard, studies have shown that older adults also adapt well to VR [61]. Unlike DS with the use of a keyboard and mouse, in the performance of VSP by older adults, VR can be advantageous, as VR is closer to actual surgery due to the immersive environment, stereoscopic vision, and bimanual working. Another point that needs to be considered is cost. Such a system would have to be acquired first. On the other hand, the use of VR, as shown in our study, is time-saving and could thereby reduce the overall cost of VSP. However, an investigation of cost was outside the scope of our study.

Trials featuring the completion of VSP in a nonfully immersive manner have been described to be very promising in the field of liver surgery [21]. However, to our knowledge, no studies have yet assessed VSP performed completely in fully immersive VR. Future studies should address, aside from segmentation, the other steps of full VSP, with the goal to perform VSP completely in VR.

Apart from the approach shown in our study, semiautomated or fully-automated segmentation algorithms are increasingly being used to accelerate or completely take over segmentation [20]. Yet, our study is concerned about which is the better working environment to perform any kind of VSP. This is even more important since the most common segmentation algorithms require a high amount of time for manual postprocessing [45], and the sole use of fully automated algorithms has still not gained general acceptance in clinical practice due to their high sensitivity to image-related artifacts, a resulting inadequate structure recognition, and a low functional stability [20]. In the future, however, artificial intelligence and VR could be combined, especially to further accelerate tasks in VR.

We not only demonstrated the feasibility of segmentation in VR but also highlighted the advantages of this. Possible explanations for these findings are the more intuitive work interface provided by the VR environment, the better spatial orientation, and the stereoscopic vision [21], which were perceived as great advantages for the purposes of VSP.

Through the crossover design, the subjects represented their own control group, and with a total of 312 experiments, the study achieved good internal validity. To improve the generalization of the results, that is, the external validity, we performed the study with subjects having different surgical experience. However, a greater diversity of subjects could lead to different results. The external validity should be evaluated in a further study, and a multicenter study is preferred to include subjects with experience from other clinics. However, this does not diminish the significance of this study. As with the various phases of drug studies, it is worthwhile to first examine the internal validity. Based on the findings of this study, a subsequent study for good external validity can be planned with more subjects but fewer examinations per subject. Furthermore, it should be considered that 2 different software programs (3D Slicer and Elucis) were compared with each other. Although both programs have a comparable command set, a slight impact cannot be excluded. However, this would not explain the large differences observed.

In contrast to the DS environment, the VR environment offers a more rapid learning curve, provides the ability to work faster, results in increased time saved, and is less exhausting when engaging in segmentation as an important substep of VSP. The VR environment was rated by participants to be easier to learn and to be their environment of choice, possibly due to its similarity to actual surgical activities. Therefore, VR-based VSP could be better integrated into clinical practice. This study can serve as a basis for investigating other common substeps of VSP. 

## Abbreviations

CAD: computer-aided design
CAS: computer-assisted surgery
CT: computed tomography
DS: desktop screen
HMD: head-mounted display
SUS: System Usability Scale
UEQ: User Experience Questionnaire
VR: virtual reality
VSP: virtual surgical planning

## Data Availability

The data presented in this study are available from the corresponding author upon reasonable request.
